# Evaluation of the utricular and saccular function using oVEMPs and cVEMPs in BPPV patients

**DOI:** 10.1186/s40463-016-0125-7

**Published:** 2016-02-09

**Authors:** Hui Xu, Fa-ya Liang, Liang Chen, Xi-cheng Song, Michael Chi Fai Tong, Jiun Fong Thong, Qing-quan Zhang, Yan Sun

**Affiliations:** Stomatology Department, Affiliated Yantai Yuhuangding Hospital of Qingdao University Medical College, Yantai City, Shandong Province China; Otorhinolaryngology Head and Neck Surgery Department, Sun Yat-sen Memorial Hospital of Sun Yat-sen University, Guangzhou, China; Otorhinolaryngology Head and Neck Surgery Department, Affiliated Yantai Yuhuangding Hospital of Qingdao University Medical College, Yantai City, Shandong Province China; Otorhinolaryngology Head and Neck Surgery Department, The Chinese University of Hong Kong, New Territories, Hong Kong, China; Otorhinolaryngology Head and Neck Surgery Department, Singapore General Hospital, Singapore, Singapore; Otology Department, Affiliated Yantai Yuhuangding Hospital of Qingdao University Medical College, Yantai City, Shandong Province China

**Keywords:** Cervical/ocular vestibular evoked myogenic potentials (c/oVEMPs), Benign paroxysmal positional vertigo (BPPV), Utricular, Saccular

## Abstract

**Background:**

It is well-known that ocular vestibular evoked myogenic potentials (oVEMPs) predominantly reflect utricular function whilst cervical vestibular evoked myogenic potentials (cVEMPs) reflect saccular function. To date, there are no published reports on the systemic evaluation of utricular and saccular function in benign paroxysmal positional vertigo (BPPV), nor are there any reports on the differences in VEMPs between patients with recurrent and non-recurrent BPPV. The aim of this study was to evaluate the difference in cervical and ocular (c/o)VEMPs between patients with BPPV and normal controls, as well as between patients with recurrent and non-recurrent BPPV.

**Methods:**

Thirty patients with posterior canal BPPV and 30 healthy subjects (as normal controls) were prospectively enrolled. cVEMP and oVEMP testing using 500 Hz tone-burst stimuli were performed on all. VEMP tests were repeated 3 times on each subject to ensure reliability and reproducibility of responses. VEMPs were defined as present or absent. Abnormal VEMP was defined by lack of VEMP response.

**Results:**

In the control group, abnormal cVEMPs responses were detected in 6.67 % and abnormal oVEMPs responses were detected in 3.34 %. In BPPV patients (10 with recurrent BPPV, 20 with non-recurrent BPPV), abnormal cVEMPs responses were detected in 30 % and abnormal oVEMPs responses were detected in 56.7 %. More patients with BPPV showed abnormal responses in c/oVEMPs as compared to the control group (*p* < 0.05). oVEMPs was more often abnormal as compared to cVEMPs in BPPV patients (*p* < 0.05). There was no statistical difference between abnormal cVEMP responses in non-recurrent BPPV patients (25 %) and recurrent BPPV patients (40 %) (*p* > 0.05). Differences in abnormal oVEMP responses (non-recurrent BPPV, 40 %; recurrent BPPV, 90 %) were significant (*p* < 0.05).

**Conclusion:**

An increased occurrence of abnormal c/oVEMP recordings appeared in BPPV patients, possibly as a result of degeneration of the otolith macula. oVEMPs were more often abnormal in BPPV patients as compared to cVEMPs, suggesting that utricular dysfunction may be more common than saccular dysfunction. Furthermore, oVEMP abnormalities in the recurrent BPPV group were significantly higher than those in the non-recurrent BPPV group. Assessment of c/oVEMPs in BPPV patients may therefore be of prognostic value in predicting likelihood of BPPV recurrence.

## Background

Vestibular evoked myogenic potential (VEMP) is a short-latency myogenic response which is evoked by brief pulses of air-conducted (AC) sound, bone-conducted (BC) vibration or electrical stimulation and recorded using surface electrodes placed over muscles. Cervical vestibular evoked myogenic potentials (cVEMPs), which are a manifestation of the vestibulo-colic reflex, predominantly the sacculo-collic reflex, are assessed by measuring electromyographic (EMG) activity from surface electrodes placed over the tonically activated sternocleidomastoid (SCM) muscles. In 1992 and 1994, cVEMPs was first described by Colebatch and Halmagyi [1, 2], who measured electromyographic (EMG) activity from the sternocleidomastoid (SCM) muscles following vestibular stimulation with brief pulses of sound (clicks). In 1995, Halmagyi et al. [3]. elicited cVEMPs by tapping the forehead with a clinical reflex hammer. The responses had the same biphasic waveform as the AC cVEMPs and were vestibular-dependent, but were also present in patients with conductive hearing loss as the stimulus bypasses the middle ear conductive mechanism. In 2000, Sheykholeslami et al. [4]. recorded cVEMPs using BC sound delivered to the mastoid bone with a clinical bone conductor.

In 2005 and 2007, Rosengren [5] and Todd [6] recorded the short latency potentials from around the eyes by bone-conducted sound (BCS) and demonstrated that it can also be recorded from the extraocular muscles as part of the vestibulo-ocular reflex (VOR). It was recently reported that ocular VEMPs (oVEMPs) are produced by synchronous activity in the extraocular muscles in response to stimulation, including sound [7]. Assessment of oVEMPs is used as a clinical test for the vestibular system because it provides information on otolith function. A more recent study reported that oVEMPs in response to air-conducted sound (ACS) reflect functions of different parts of the vestibular labyrinth from cVEMPs in response to ACS; that is, oVEMPs predominantly reflect utricular functions while cVEMPs reflect saccular functions [8].

Canalolithiasis and cupulolithiasis have been considered as possible mechanisms in the etiology of benign paroxysmal positional vertigo (BPPV) [9, 10]. In addition to these mechanisms, otolith dysfunction has also been suggested as a possible mechanism of BPPV [11–13]. The detachment of the otoconia from the otolith macula is post-viral or post-traumatic in some cases; however, in many instances it seems to occur without an identifiable cause [14]. The finding that BPPV patients had significantly higher incidence of abnormal amplitudes in cVEMPs compared with controls has been reported [15, 16], suggesting that BPPV patients have more saccular damage than controls. However, the systemic evaluation of utricular and saccular damage in BPPV patients has never been reported. There is a significant rate of BPPV recurrence after initial resolution. The reported recurrence rate during a 1-year follow-up period ranged from 10 % to 18 % [17, 18]. To date, there are no reports on the differences in VEMPs between patients with recurrent and non-recurrent BPPV. The aim of this study is to evaluate the difference in c/oVEMPs between patients with BPPV and controls, as well as between patients with recurrent and non-recurrent BPPV. The other objective of this study is to compare oVEMP and cVEMP results in patients with BPPV.

## Methods

### Subjects

Ethical approval was received from the Ethics Committee of Affiliated Yantai Yuhuangding Hospital of Qingdao University Medical College. We prospectively enrolled 30 consecutive patients from the Dizziness Clinic, affiliated Yantai Yuhuangding Hospital of Qingdao University Medical College, who were diagnosed with posterior canal BPPV (canalolithiasis, nystagmus duration < 60 s) between January 2013 and June 2014. Follow-up was for 1 year. Patients were divided into two groups according to recurrence - 20 non-recurrent BPPV patients and 10 recurrent BPPV patients. Recurrence was defined as BPPV that occurred more than 1 month after a successful repositioning manoeuvre during the 1 year follow-up period. The diagnosis of BPPV and the affected side was based on the typical nystagmus seen during the Dix-Hallpike maneuver. The control subjects were all volunteers from our normal outpatient clinic who had no otological disease. Patients with a history of hearing loss, other vestibular disorders and >60 years old were excluded. Informed consent was obtained from each subject according to the Declaration of Helsinki.

### cVEMPs

Cervical vestibular evoked myogenic potentials (cVEMPs) testing was performed on both sides for all patients and controls. In the cVEMPs test, all subjects were placed in a sitting position and asked to rotate their head away from the stimulated side so as to record electromyographic activity over tonically activated sternocleidomastoid (SCM) muscles. Surface EMG activity was recorded with superficial electrodes placed on the middle third of the SCM, with the reference electrode placed on the upper third of the sternum and the ground electrode on the middle of the forehead. Using a Bio-Logic Navigator Pro, 90 dB nHL 500 Hz tone bursts were presented through headphones, and the EMG signal was amplified and bandpass filtered (30–1500 Hz). The analysis window was 100 ms wide and responses to 120 stimuli were averaged. cVEMP tests were repeated 3 times on each subject to ensure reliability and reproducibility of responses. The amplitude of the first positive–negative peak (P13–N23) was recorded. Absence of a meaningful wave form with p13 and n23 was defined as ‘no response’. Abnormality was strictly defined as a cVEMP pattern of ‘no response,’ which meant the absence of a meaningful waveform with P13 and N23.

### oVEMPs

Ocular vestibular evoked myogenic potentials (oVEMPs) testing was performed on both sides for all patients and controls. In the oVEMPs test, all subjects assumed a sitting position and the subject was instructed to look superomedially at a small fixed target 1 m from the eyes. The visual angle was approximately 30°, which has been found to elicit the largest responses compared with other eye positions [19]. The active electrodes were placed on the face, oriented vertically and approximately 1 cm below the center of the lower eyelid just inferior to the contralateral eye for sound stimulation. The reference electrode was placed about 1 cm below the active electrode on the cheek, and the ground electrode was placed on the forehead. Each subject’s eyes remained fixed on the target throughout the test. Using a Bio-Logic Navigator Pro, 95 dB nHL 500 Hz tone bursts were presented through headphones, and the EMG signal was amplified and bandpass filtered (10–300 Hz). The analysis window was 100 ms wide and responses to 120 stimuli were averaged. oVEMP tests were repeated 3 times on each subject to ensure reliability and reproducibility of responses. The initial negative–positive biphasic waveform comprised peaks N1 and P1. We analyzed the waveforms of N1 and P1 at the maximal intensity of stimulation. Abnormality was strictly defined as an oVEMP pattern of ‘no response,’ which meant an absence of a meaningful waveform with N1 and P1.

### Statistical analysis

A Fishers exact or Chi-squared test was used to analyze the statistical significance of the inter-group difference in the number of non- cVEMPs and oVEMPs responders in the BPPV and controls groups. A *p* value of < 0.05 indicated statistical significance.

## Results

The ages ranged from 34 to 55 years (mean 45.5, 12 men and 18 women) in patients with BPPV. The control group consisted of 30 normal subjects (10 men and 20 women; mean age 42.2 years; age range 30–60 years). Demographic data for BPPV and control groups are summarized in Table 1. There were no significant differences in age and sex ratio between the two groups (*p* > 0.05). Testing of VEMPs was performed on both sides in all BPPV patients and controls. Only one patient with BPPV showed bilateral abnormalities, so only ipsilesional data are presented. In the control group, abnormal cVEMP responses were detected in 2 of 30 (6.67 %) subjects and abnormal oVEMP responses were detected in 1 of 30 (3.34 %) subjects (Table 2).

**Table 1:** Demographic features of subjects in BPPV and control groups

Group feature	Control	BPPV
Number	30	30
Age*	42.2 ± 8.7	45.5 ± 9.2
Sex (M:F)*	10:20	12:18

*BPPV* Benign paroxysmal positional vertigo. *p > 0.05

**Table 2:** The abnormal c/oVEMP responses details in control group

VEMP test	Abnormal	Normal	Total	Percent
cVEMP	2	28	30	6.67 %
oVEMP	1	29	30	3.34 %

### cVEMP abnormalities in BPPV patients

**Fig. 1:**
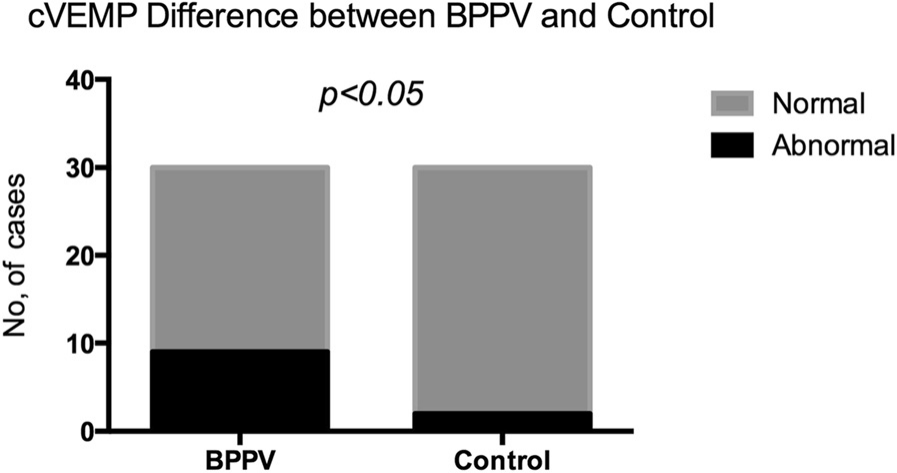
cVEMP abnormalities difference between BPPV and control groups. Legend 1: Abnormal cVEMPs responses were detected in 30 % BPPV subjects. In control volunteers, abnormal cVEMPs responses were detected in 6.67 % subjects. More of the patients with BPPV showed abnormal responses in cVEMPs than the controls (*p* < 0.05)

**Fig. 2:**
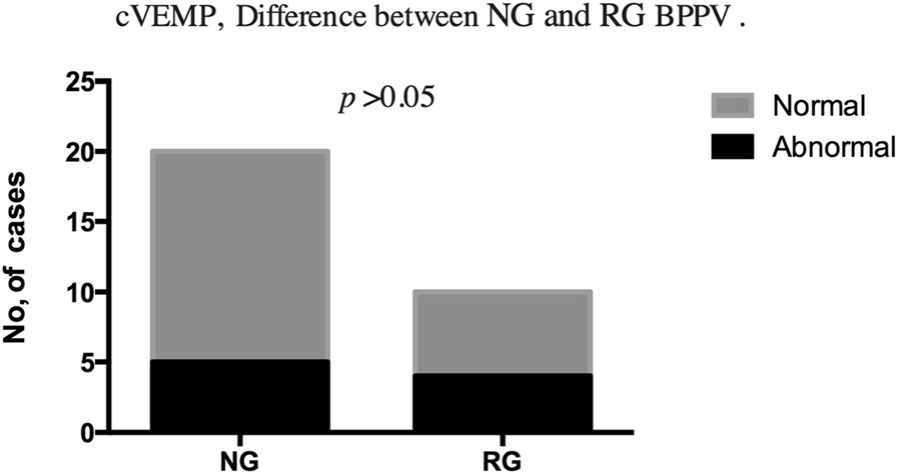
cVEMP abnormalities difference between nonrecurrent and recurrent BPPV groups. Legend 2: In cVEMPs testing, abnormalities were detected in 25 % in the nonrecurrent BPPV group and in 40 % subjects in the recurrent BPPV group; the difference between the two groups was no significant (*p* > 0.05). (*NG* Nonrecurrent group, *RG* Recurrent group)

Abnormal cVEMP responses were detected in 9 of 30 (30 %) BPPV subjects (Table 3). More patients with BPPV showed abnormal responses in cVEMPs as compared to the controls (*p* < 0.05) (Fig. 1). In cVEMPs testing, abnormalities were detected in 5 of 20 (25 %) in the non-recurrent BPPV group and in 4 of 10 (40 %) subjects in the recurrent BPPV group; the difference between the two groups was not significant (*p* > 0.05) (Fig. 2).

**Table 3:** cVEMPs abnormalities in BPPV groups

BPPV group	Abnormal	Normal	Total
NG	5 (25 %)	15 (75 %)	20
RG	4 (40 %)	6 (60 %)	10
Total	9 (30 %)	21 (70 %)	30

*NG* Nonrecurrent group, *RG* Recurrent group, NG VS RG: (*p* > 0.05)

### oVEMP abnormalities in BPPV patients

**Fig. 3:**
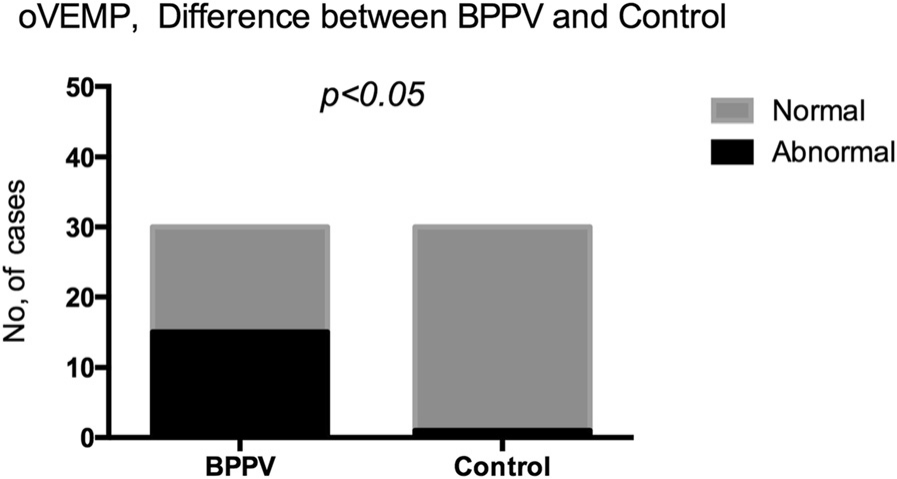
oVEMP abnormalities difference between BPPV and control groups. Legend 3: In control volunteers, abnormal oVMEPs responses was detected in 3.34 % subjects. Abnormal oVMEP responses were detected in 56.7 % subjects in BPPV group. More of the patients with BPPV showed abnormal responses in oVEMPs than the controls (*p* < 0.05)

**Fig. 4:**
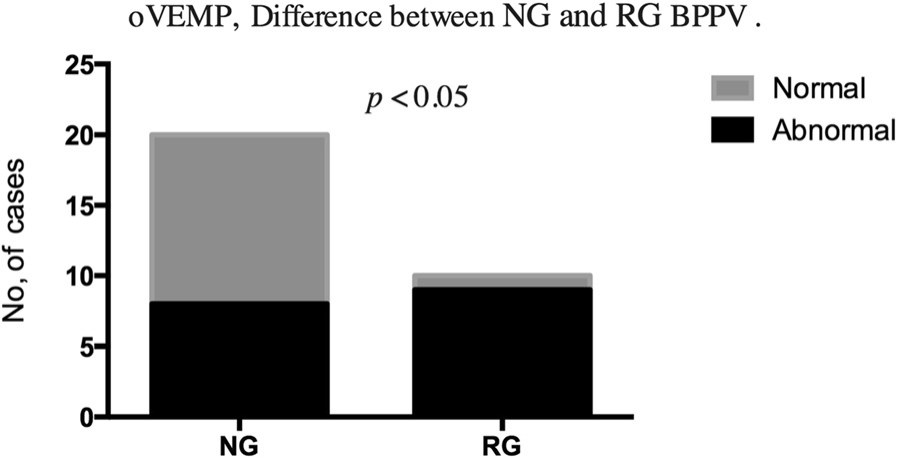
oVEMP abnormalities difference between nonrecurrent and recurrent BPPV groups. Legend 4: In oVEMPs testing, abnormalities were detected in 40 % in the nonrecurrent BPPV group and in 90 % subjects in the recurrent BPPV group; the difference between the two groups was significant (*p* < 0.05). (*NG* Nonrecurrent group, *RG* Recurrent group)

Abnormal oVEMP responses were detected in 17 of 30 (56.7 %) subjects in BPPV group (Table 4). More patients with BPPV showed abnormal responses in oVEMPs as compared to the controls (*p* < 0.05) (Fig. 3). In oVEMPs testing, abnormalities were detected in 8 of 20 (40 %) in the non-recurrent BPPV group and in 9 of 10 (90 %) subjects in the recurrent BPPV group; the difference between the two groups was significant (*p* < 0.05) (Fig. 4).

**Table 4:** oVEMP abnormalities in BPPV groups

BPPV group	Abnormal	Normal	Total
NG	8 (40 %)	12 (60 %)	20
RG	9 (90 %)	1 (10 %)	10
Total	17 (56.7 %)	13 (43.3 %)	30

*NG* Nonrecurrent group, *RG* Recurrent group; NG VS RG: (*p* < 0.05)

### Comparison of oVEMPs with cVEMPs within BPPV patients

**Fig. 5:**
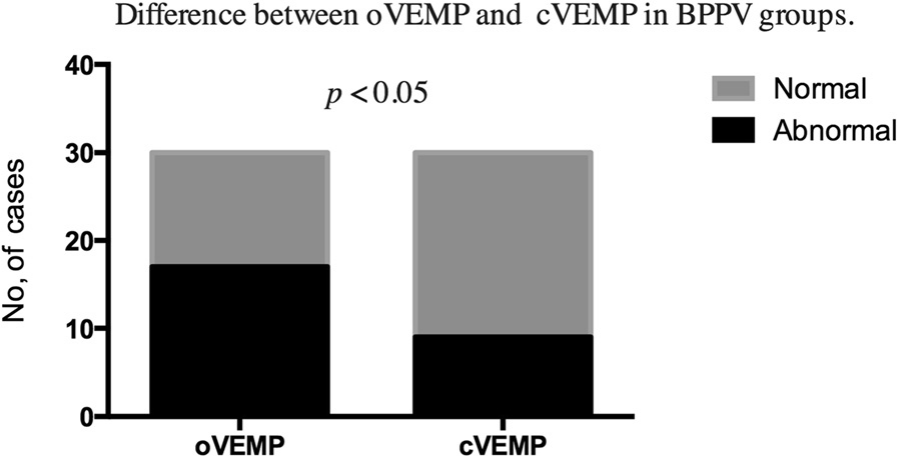
Difference between oVEMP and cVEMP in BPPV groups. Legend 5: Abnormal oVMEPs responses were detected in 56.7 % subjects while abnormal cVMEPs responses were detected in 30 % subjects in BPPV group. The abnormal results for oVEMPs showed a higher percentage than those for cVEMPs in BPPV patients (*p* < 0.05)

Abnormal oVMEPs responses were detected in 17 of 30 (56.7 %) subjects in BPPV group while abnormal cVMEPs responses were detected in 9 of 30 (30 %). The abnormal results for oVEMPs showed a higher percentage than those for cVEMPs in BPPV patients (*p* < 0.05) (Fig. 5).

## Discussion

The exact pathophysiology of BPPV remains unclear. Several studies have previously suggested the cause being dislodgement of otoconia from the gelatinous layer of the otolithic membrane [20], which may be associated with osteopenia and osteoporosis [21]. At present, cVEMPs which are evoked by air-conducted sound (ACS), are widely used to evaluate the function of saccule and inferior vestibular nerve by recording the inhibitory potential from the SCM. oVEMPs are also evoked by ACS. Although its origin is controversial, it was recently reported to be involved in the stimulation of the utricular macula. Curthoys et al. [7]. and Shin et al. [22]. reported that the oVEMP evoked by ACS may be predominantly mediated by the superior vestibular nerve due to the activation of the utricular receptors. In BPPV, the degenerative process of otolith not only affects the macula of the utricle and causes detachment of the otoliths, but might also affect the macula of the saccule. Von Brevern et al. [23]. reported that utricular dysfunction, which is associated with idiopathic BPPV, possibly results from degeneration of the utricular macula. Our study results supports this hypothesis with regards to utricular dysfunction in BPPV patients. However, they failed to demonstrate any significant change in saccular function. Our study is in concordance with several other studies that reported on cVEMPs in patients diagnosed with BPPV [15, 24]. Korres S et al. [24]. found an increased occurrence of abnormal cVEMP recordings in BPPV patients and attributed this to possible degeneration of the saccular macula, which is part of the neural VEMP pathway. With regards to the amplitude and latency values of VEMPs, there are many factors that can affect these values such as basic muscle activity, patient’s position, and general conditions [25, 26]. Because of their non-specific value in VEMPs testing, we used a qualitative approach to VEMPs results. Our definition for abnormal VEMP is an absence of VEMP response. The main objective in this study was to report c/o VEMPs findings in BPPV patients and to verify some clinical characteristics of BPPV in VEMPs. We evaluated both the function of utricle and saccule by measuring c/oVEMPs.

In our study, we found that patients with BPPV showed higher rate of abnormal responses in c/oVEMPs by stimulation on their affected side than the controls. Hong et al. 16 reported that 24.5 % of patients with BPPV showed abnormal cVEMP responses, such as P13 latency prolongation and VEMP amplitude asymmetry on the affected side. The incidence of abnormal response of cVEMP in our study was 30 %, which is similar with that reported. Brandt et al. [27]. reported that most recurrences were diagnosed within the first year after treatment. Hence, 1 year was chosen as the follow up period in our study. In cVEMPs testing, abnormalities were detected in 25 % of subjects in the non-recurrent BPPV group and in 40 % of subjects in the recurrent BPPV group. Although there was a higher rate of abnormality in the recurrent BPPV group, the difference between the two groups was not significant (*p* > 0.05). This suggests that BPPV recurrence may not be related to saccular damage, although another possibility may be that larger numbers of patients are needed in the study to demonstrate a significant difference. Our findings differ from those of Lee JD et al., who reported a higher incidence (31.25 %) of cVEMP abnormality in recurrent BPPV than non-recurrent BPPV patients [28].

In oVEMPs testing, abnormal response rate was significantly higher in recurrent BPPV patients than non-recurrent BPPV patients, whilst the difference in cVEMPs between the 2 groups was not significant. This suggests that the incidence of utricular dysfunction is higher than saccular dysfunction in recurrent BPPV patients. Clinicians should be aware of the risk of recurrent BPPV in these patients with abnormal oVEMPs and be able to counsel patients appropriately. In our clinical practice, the risk of recurrence is emphasized after the diagnosis of BPPV and strict follow up is recommended for all patients. With our findings, we may now be able to better predict which patients are more likely to have BPPV recurrence.

In our study, there was a higher rate of abnormal oVEMP results than cVEMP results in BPPV patients. These findings support the possibility that utricular function in BPPV patients is more heavily damaged than saccular function. Utricular dysfunction seems to play an essential role as an underlying mechanism contributing to BPPV. Bremova T et al. [29]. measured oVEMP amplitudes before and after repositioning manoeuvres in BPPV patients and found significant increase in the amplitudes following the manoeuvres whilst the cVEMPs had no amplitude change.

VEMPs is a good method for evaluation of otolith function; however, this test requires specialized equipment and complicated procedures for separate analyses of utricular and saccular function as the VOR gain reduction is under the influence of both utricular and saccular dysfunction, in addition to semicircular canal-otolith interaction. In the present study, abnormalities in oVEMPs was frequently detected. This finding is consistent with the suggested underlying pathology of BPPV, namely, degeneration of the utricular maculae leading to dislodging of otoconia. Those ears showing abnormal oVEMP as well as cVEMP might have severe changes causing dysfunction of the utricle and the saccule.

Considering the controversy in the stability and repeatability of c/oVEMPs, we defined abnormal c/oVEMPs as absent responses in our study. As a result, we did not analyse the latency and amplitude of the waves in c/oVEMPs. The main limitation of our study is the small number of BPPV patients. As the incidence of BPPV is higher in the elderly and the average age of our study patients was under 60 years, there is a potential bias in the research. In future research, a larger sample size will be obtained such that quantitative analysis of otolith function in BPPV patients can be performed.

## Conclusion

An increased occurrence of abnormal c/oVEMP recordings appeared in BPPV patients, possibly as a result of degeneration of the otolith macula. oVEMPs were more often abnormal in BPPV patients as compared to cVEMPs, suggesting that utricular dysfunction may be more common than saccular dysfunction. Furthermore, oVEMP abnormalities in the recurrent BPPV group were significantly higher than those in the non-recurrent BPPV group. Assessment of c/oVEMPs in BPPV patients may therefore be of prognostic value in predicting likelihood of BPPV recurrence in these patients.
